# Exploratory integration of near-infrared spectroscopy with clinical data: a machine learning approach for HCV detection in serum samples

**DOI:** 10.3389/fmed.2025.1596476

**Published:** 2025-06-09

**Authors:** Eloy Pérez-Gómez, José Gómez, Jennifer Gonzalo, Sergio Salgüero, Daniel Riado, María Luisa Casas, María Luisa Gutiérrez, Elena Jaime, Enrique Pérez-Martínez, Rafael García-Carretero, Javier Ramos, Conrado Fernández-Rodríguez, Myriam Catalá, Luca Martino, Óscar Barquero-Pérez

**Affiliations:** ^1^Department of Signal Theory and Communications, EIF, University Rey Juan Carlos, Fuenlabrada, Spain; ^2^Department of Biology and Geology, Physics and Inorganic Chemistry, ESCET, University Rey Juan Carlos, Móstoles, Spain; ^3^Instituto de Investigación en Cambio Global (IICG-URJC), Universidad Rey Juan Carlos, Móstoles, Spain; ^4^Service of Clinical Biochemistry, Hospital Universitario Fundación Alcorcón, Alcorcón, Spain; ^5^Service of Gastroenterology, Hospital Universitario Rey Juan Carlos, Fuenlabrada, Spain; ^6^Service of Gastroenterology, Hospital Universitario Fundación Alcorcón, Alcorcón, Spain; ^7^Hospital Universitario Mostoles, Móstoles, Spain; ^8^Department of Medical Specialties and Public Health, University Rey Juan Carlos, Alcorcón, Madrid, Spain; ^9^Dipartimento di Economia e Impresa, Universita di Catania, Catania, Italia

**Keywords:** NIRS, HCV, Hepatitis C, machine learning, permutation feature importance

## Abstract

**Background:**

Managing chronic viral infections like Hepatitis C virus (HCV) often requires expensive healthcare resources and highly qualified personnel, making efficient diagnostic methods essential. Despite remarkable therapeutic advancements for the treatment of HCV, several challenges remain, such as improved fast diagnostic procedures allowing universal screening.

**Objective:**

We propose a novel approach that combines Near-Infrared Spectroscopy (NIRS) and clinical data with machine learning (ML) to improve Hepatitis C Virus (HCV) detection in serum samples.

**Methods:**

NIRS offers a fast, non-destructive, and residue-free alternative to traditional diagnostic methods, while ML models enable feature selection and predictive analysis. We applied L1-regularized Logistic Regression (L1-LR) to identify the most informative wavelengths for HCV detection within the 1,000–2,500 nm range, and then integrated these spectral features with routine clinical markers using a Random Forest (RF) model. Our dataset comprised 137 serum samples from 38 patients, each represented by a NIRS spectrum and clinical data from blood tests.

**Results:**

After preprocessing with Standard Normal Variate (SNV) correction and downsampling, the best-performing RF model, which combined NIRS features and clinical data, achieved an accuracy of 72.2% and an AUC-ROC of 0.850, outperforming models using only clinical or spectral data. Feature importance analysis highlighted specific wavelengths near 1,150 nm, 1,410 nm, and 1,927 nm, associated with water molecular states and liver function biomarkers (GPT, GOT, GGT), reinforcing the biological relevance of this approach.

**Conclusions:**

These findings suggest that integrating NIRS and clinical data through machine learning enhances HCV diagnostic capabilities, offering a scalable and non-invasive alternative for early detection and risk assessment.

## 1 Introduction

The use of rapid diagnostic tools, as demonstrated during the COVID-19 pandemic, is crucial for preventing the rapid spread of infectious diseases. These tools must be both cost-effective and reliable, particularly in large-scale applications. Virus-induced diseases, such as Hepatitis C virus (HCV) infections, further underscore their importance. Managing chronic infections like HCV often requires expensive healthcare resources and highly qualified personnel, making efficient diagnostic methods essential. Despite remarkable therapeutic advancements for the treatment of HCV, several challenges remain, such as improved fast diagnostic procedures allowing universal screening (1, 2), and the need for better models to stratify hepatocellular carcinoma (HCC) risk (3).

Near Infrared Spectroscopy (NIRS) is a promising alternative to other diagnosis methods, such as gas chromatography, high-performance liquid chromatography (HPLC) or PCR, as all these methods are time-consuming, destructive and produce contaminating residues (4). NIRS analyzes a sample by irradiating it with light and measuring its absorption spectrum between 780 and 2,500 nm. The resulting spectrum consists of overlapping absorption responses from various functional groups and molecules present in the sample. Each of these responses is characteristic of a specific molecular composition, forming what is known as the global molecular fingerprint (GMF) (5). This unique spectral signature, GMF, allows NIRS to differentiate samples with distinct characteristics.

Biological samples are often found in aqueous solutions, where water strongly absorbs in the NIRS range, influencing the absorption patterns of other molecules in the sample. However, the molecular structure of water itself exhibits specific spectral patterns that reflect the composition of the surrounding solution. Aquaphotomics is the study of these water-related spectral patterns, providing valuable insights into the composition of biological samples and enhancing the accuracy of NIRS analysis (6).

Machine learning has become an essential tool for analyzing NIRS data, due to its ability to effectively manage complex, high-dimensional datasets and enhance predictive accuracy. Over time, various machine learning algorithms, ranging from traditional methods to sophisticated deep learning models, have been applied to advance the analysis of NIRS data (7). In agriculture and food quality, the combination of NIRS and machine learning techniques has been instrumental in characterizing products like rice, mapping properties such as glycemic index and amylose content to spectral data (8, 9). Similarly, in environmental monitoring, the combination of machine learning and NIRS aids in the rapid assessment of water pollution, with machine learning models like support vector machines improving the prediction of pollution indicators (10).

The use of machine learning techniques and NIRS offers significant potential in medical diagnostics. When combined with machine learning, NIRS enhances the detection and classification of various medical conditions by analyzing complex spectral data. For example, it has been employed to rapidly determine hemoglobin concentration using a novel ensemble extreme learning machine method, demonstrating its ability to provide quick, non-invasive diagnostics (11). In oncology, NIRS, along with machine learning algorithms, like support vector machines (SVMs), has been applied to detect neutropenia, potentially reducing the need for invasive tests (12). In the pharmaceutical industry, NIRS combined with machine learning algorithms such as deep belief networks and extreme learning machines is used to classify and identify drugs, ensuring patient safety and improving drug quality control (13). Moreover, NIRS combined with machine learning has been tested in the early diagnosis of Type 2 diabetes, as demonstrated in studies where aquaphotomics aids in identifying biomarkers for the disease (14). A preclinical proof of concept demonstrated the effectiveness of this tandem in fast viral detection, showcasing its potential for early diagnosis and quick clinical decision-making. Specifically, in the case of Hepatitis C, NIRS, coupled with L1-penalized classification algorithms, has been successfully employed to predict HCV positivity from serum microsamples (15, 16).

In this study, we present a proof of concept to explore the integration of NIRS, machine learning, and clinical features from routine blood tests for HCV detection. Our primary objective is to assess whether this multimodal approach can provide meaningful insights and serve as a foundation for more advanced applications, such as predicting HCC risk. By leveraging the rapid, non-destructive nature of NIRS alongside machine learning-driven analysis of spectral and clinical data, we aim to address key challenges in HCV diagnostics, including the need for faster, cost-effective screening methods and improved risk stratification models for HCC. Our approach seeks to evaluate whether integrating these diverse data sources can enhance diagnostic capabilities, ultimately contributing to more accessible and scalable screening strategies, trying to address the remaining challenges in the management of HCV. To our knowledge, this is the first attempt to combine NIRS, machine learning, and clinical features for HCV detection, offering a novel perspective on their synergy. If successful, this methodology could serve as a stepping stone for broader applications in predictive medicine, particularly in early disease detection and prognosis.

To achieve this, we must effectively extract relevant information from the complex spectral data. The spectrum obtained from a sample using NIRS contains a vast amount of information, presenting a high-dimensionality challenge from a machine learning perspective. In such cases, dimensionality-reduction and feature selection techniques play a crucial role in identifying the most relevant features for classification while enhancing model interpretability and generalization. We propose to use L1-regularized Logistic Regression (LR) as an initial step to reduce the number of variables and identify the most informative wavelengths for HCV detection. Additionally, we will implement multiple feature selection techniques, including permutation importance combined with ensemble-based methods, to further refine the feature set, ensuring that the final model remains parsimonious while retaining predictive power.

The remainder of the manuscript is organized as follows. Section 2 describes the dataset used in this study, including its sources and characteristics. Section 3 introduces the machine learning models employed, detailing their architectures and relevance to the problem at hand. Section 4 outlines the feature importance methods used to interpret the models and identify key features. Section 5 presents the experimental setup, including evaluation metrics, training procedures, and validation strategies. Section 6 discusses the results, highlighting the performance of the models and the insights gained from feature importance analysis. Section 7 provides a discussion of the findings, their implications, and potential limitations, and a summary of the key contributions and suggesting directions for future research.

## 2 Dataset

A total of 137 serum samples from 38 HCV patients were randomly selected from the HCV biobank collection at the Hospital Universitario Fundación de Alcorcón (HUFA) Biobank. These samples were obtained between years 2000 and 2010. All patients were initially treated with peginterferon plus ribavirin, as serum samples were collected prior to the approval of direct-acting antivirals (DAAs) in Spain in 2014. All non-responders were subsequently rescued with DAAs. HCV infection was confirmed using polymerase chain reaction (PCR) for HCV-RNA detection, which is considered the gold standard for confirming active HCV infection. Quantification of HCV RNA was performed using the real-time PCR assay Roche COBAS 4800, following the manufacturer's protocol. The assay has a lower limit of detection (LOD) of 7.6 IU/ml and a quantification range of 15 to 10^8^ IU/ml (1.2–8.0 Log IU/ml), and the lower limit of quantification (LLOQ) is 15 IU/ml (17).

In addition to the serum samples, patient-associated clinical and biochemical features were also available, including sex, albumin levels (g/dl), international normalized ratio (INR), platelet count (10^3^/*mcL*), creatinine (mg/dl), bilirubin (mg/dl), alanine transaminase (GPT, U/L), aspartate transaminase (GOT, U/L), alkaline phosphatase (FAL, U/L), gamma-glutamyl transferase (GGT, U/L), and age. Serum aliquots from the biobank were thawed at room temperature, and 70 μl were carefully transferred to sterile, hermetic borosilicate glass vials under a biosafety hood by qualified biobank personnel. The aliquots were then preserved on ice or refrigerated at temperatures below 4°C until spectral acquisition. After this, they were ultrafrozen at −80°C to ensure sample integrity for long-term storage (18).

### 2.1 Ethical considerations

The study was approved by the HUFA Ethics Committee for Research with Medicines (CEIm), Approval Number 20-208 22/12/2020, which authorized the project and granted a waiver of informed consent for the use of biobank samples. This exemption complied with national regulations and international ethical standards, including the World Medical Association's Declaration of Helsinki.

Informed consent was waived because all serum samples had been collected prior to the implementation of national legislation requiring explicit consent for secondary use of biological materials. At the time, sample collection followed the clinical and institutional protocols in place, which did not mandate individual consent for future anonymized research. All samples were fully anonymized before analysis, and no clinical or identifying data were accessible to the investigators. The ethics committee considered the historical context and the absence of foreseeable risk to participants in granting this approval, ensuring compliance with all applicable legislation.

### 2.2 Acquisition of FT-NIRS spectra

Fourier Transform Near-Infrared Spectroscopy (FT-NIRS) spectra were acquired using a Spectrum 100 N spectrophotometer (PerkinElmer, Beaconsfield, UK) with Spectrum software (version 6.3.4). Samples were maintained at 37°C using an Accublock^TM^ thermoblock, with temperature cross-checked regularly by an independent thermometer to ensure accuracy. Spectral measurements were performed in reflectance mode, capturing data between 1,000 and 2,500 nm at a resolution of 0.5 nm. Each spectrum represented an average of 64 consecutive measurements. An empty vial served as the reference for baseline correction.

Table 1 summarizes the dataset, presenting the average and standard deviation for each clinical feature, total and disaggregated for HCV positive (detectable) and negative (undetectable) serum samples. Last column shows univariate *t*-test using the presence of the virus in the sample as independent variable.

**Table 1:** Summary of clinical features.

**Feature**	**Total (*n* = 106)**	**Undetectable HCV (*n* = 62)**	**Detectable HCV (*n* = 44)**	***p*-Value**
Sex	F (33), M (73)	F (16), M (46)	F (17), M (27)	–
Albumin (g/dl)	4.25 ± 0.29	4.25 ± 0.28	4.25 ± 0.31	0.99
INR	1.03 ± 0.09	1.02 ± 0.09	1.04 ± 0.08	0.17
Platelet (10^3^/mcl)	162.24 ± 60.15	168.05 ± 62.62	154.05 ± 56.17	0.23
Creatinine (mg/dl)	1.02 ± 0.10	1.02 ± 0.10	1.02 ± 0.10	0.91
Bilirubin (mg/dl)	0.77 ± 0.35	0.78 ± 0.38	0.75 ± 0.31	0.71
GPT (U/L)	42.35 ± 33.87	28.31 ± 22.29	62.14 ± 37.57	<0.001*
GOT (U/L)	33.70 ± 21.06	25.71 ± 16.33	45.21 ± 21.93	<0.001*
FAL (U/L)	92.03 ± 51.54	93.08 ± 52.88	90.55 ± 50.15	0.80
GGT (U/L)	34.20 ± 31.38	26.02 ± 26.69	46.00 ± 34.08	0.002
Age	61.16 ± 10.35	59.13 ± 7.90	64.02 ± 12.60	0.03

Data are shown as mean ± standard deviation. Last column shows univariate *t*-test using the presence of the virus in the sample as independent variable. Sex (F: Female, M: Male). ^*^ indicates statistical significancy (*p* < = 0.05).

## 3 Machine learning models

This section provides an overview of the machine learning models used to analyze the NIRS data for detecting HCV in serum samples. It begins with preprocessing techniques to correct scattering effects and to reduce dimensionality in the NIRS data, followed by the description of the machine learning used to predict the presence of HCV, in particular, simple LR for initial model selection, the use of *l*1-penalized LR for feature selection and concludes with the integration of selected features and clinical data using Random Forest (RF) to enhance prediction accuracy.

In this work, each sample is represented as the pair (***x***_*i*_, *y*_*i*_), where ***x***_*i*_ is a feature vector containing the 3,001 features (wavelengths), and *y*_*i*_ indicates the presence (*y*_*i*_ = 1) or absence (*y*_*i*_ = 0) of HCV in the serum. In the final model, we integrate the clinical data, so each sample would be represented as the pair $\left({\text{x}}_{i}^{*},y_{i}\right)$, where ${\text{x}}_{i}^{*}$ is an extended vector with NIRS data and clinical features, with a dimensionality (without any feature selection) of 3012.

### 3.1 Preprocessing

Preprocessing of NIRS data is an important step, aimed at minimizing the influence of physical phenomena that can obscure the true chemical information within spectra. These interferences, arising from instrumental noise, light scattering, temperature fluctuations, variations in particle size, and path length inconsistencies, can compromise the assumptions underlying the classification models. To enhance model robustness and improve data reliability, preprocessing techniques are typically categorized into scatter-correction methods and spectral derivatives. In this study, we are going to test two widely adopted scatter-correction methods: Multiplicative Scatter Correction (MSC) and Standard Normal Variate (SNV). Both approaches are designed to mitigate the effects of light scattering and baseline shifts, ensuring that the spectral data accurately represent the chemical composition of the samples. By applying these methods, we aim to improve the signal-to-noise ratio and enable the development of more accurate and reliable models (19–21).

Assuming that an observed NIRS spectrum, *x*, as a function of the wavelength, can be represented as:


$$x\left(\lambda \right)=\alpha A_{0}\left(\lambda \right)+\beta +n\left(\lambda \right)$$

where, λ represents the wavelength, *A*_0_(λ) is the real spectrum, α is a multiplicative scatter factor, β is an additive scatter factor, and *n*(λ) is additive noise function of λ. Then, the MSC and SNV preprocessing methods can be described as follows:

- **MSC:** Is a method that tries to estimate the multiplicative and additive scatter factors (α, β) using a linear regression model. This technique requires a reference spectrum, i.e. an estimation of the real spectrum Â_0_(λ), ideally free of scattering effects. The maximum likelihood estimator (the average spectrum) is used as reference, assuming the scattering effects for each realization are approximately white additive, ${Â}_{0}\left(\lambda \right)=1/N\underset{i}{\sum }x_{i}\left(\lambda \right)$. So, for each NIRS data spectrum, the scattering factors, α and β, are estimated using least squares and then the spectrum is corrected:
$$x_{i}\left(\lambda \right)\approx {\hat{\alpha }}_{i}+{\hat{\beta }}_{i}{Â}_{0}\left(\lambda \right)$$
$$x_{i}^{MSC}\left(\lambda \right)=\frac{x_{i}\left(\lambda \right)-{\hat{\alpha }}_{i}}{{\hat{\beta }}_{i}}$$
- **SNV:** It is a method that allows to correct vertical baseline drift. It centers and standardizes each spectrum with respect to its mean and standard deviation, so intensities after SNV has zero mean and 1 standard deviation.
$$x_{i}^{SNV}=\frac{x_{i}\left(\lambda \right)-{\bar{x}}_{i}}{\sigma_{i}}$$
  where, ${\bar{x}}_{i}$, and σ_*i*_ are the mean and standard deviation of the spectrum, respectively.

NIRS equipment has a high resolution (0.5 nm), meaning each spectrum has information from 3,001 wavelengths (from 1,000 to 2,500 nm), which could be highly redundant. While this redundancy ensures that all relevant spectral information is captured, it can pose challenges when using machine learning models. First, it results in input features that are highly correlated, which can lead to multicollinearity issues. Additionally, the high dimensionality of the feature space can increase computational complexity and the risk of overfitting, making dimensionality reduction techniques necessary for efficient modeling. We propose to explore, first, whether using **downsampling techniques** will allow us to reduce the dimensionality while keeping the prediction capabilities of machine learning methods. We need to choose which sample rate we will use to reduce the dimension of the problem while keeping all the information needed to produce adequate results. We will explore several downsampling rates: 2, 4, 8, 16, and 25; based on information from the average mutual information from the NIRS spectra.

In order to deal with missing values when incorporating clinical data in the machine learning model, we propose to use the median to impute the data.

### 3.2 Machine learning models

We propose a multi-stage machine learning pipeline to optimize data preprocessing, feature selection, and predictive model performance. Initially, we assessed various preprocessing techniques using a baseline machine learning model, in particular a LR model to determine their feasibility and impact on downstream analysis. Next, we employed an L1-regularized LR to perform feature selection on NIRS by promoting sparsity in model coefficients, thereby identifying the most informative wavelengths. Finally, we integrated these selected spectral features with available clinical data and using a RF model to exploit both spectral and clinical information for enhanced predictive accuracy. This approach enabled a systematic refinement of the input feature space while leveraging complementary data modalities to improve model robustness and interpretability.

#### 3.2.1 Logistic regression

LR is a binary classifier that models the probability of a given input ***x***, (in our problem the NIRS values) belonging to a class *y* = 1 against class *y* = 0. It is based on the logistic, or sigmoid, function, which transforms a linear input (linear combination of the input features, ***x***) into a value between 0 and 1, interpreted as a probability of belonging to class *y* = 1:


(1) $$\begin{array}{c} P\left(y=1|x\right)=\sigma \left({\text{x}}^{T}\beta \right)=\frac{1}{1+e^{-\left({\text{x}}^{T}\beta \right)}}, \end{array}$$

where σ is the sigmoid function, β is the vector of coefficients associated with the vector of features ***x***. These β coefficients are estimated by minimizing a cost function (usually cross-entropy) that measures the mismatch between the predicted class ŷ_*i*_ and the real class *y*_*i*_:


$$\begin{array}{c} J\left(\beta \right)=-\frac{1}{m}\overset{m}{\underset{i=1}{\sum }}\left[y_{i}\log\left({ŷ}_{i}\right)+\left(1-y_{i}\right)\log\left(1-{ŷ}_{i}\right)\right], \end{array}$$

where *m* is the number of training samples.

#### 3.2.2 L1-regularized logistic regression

To improve the generalization of LR models and prevent overfitting, regularization techniques are introduced to penalize overly complex models and encourage simpler, more robust solutions. This is achieved by incorporating the norm of the model coefficients into the objective function *J*(***β***), thereby discouraging excessively large coefficient values (22, 23).

We propose to use **L1-regularized LR**, which adds a penalty equal to the sum of the absolute values (L1 norm) of the coefficients. The cost function is then:


$$\begin{array}{c} J_{L_{1}}\left(\beta \right)=J\left(\beta \right)+\lambda \overset{n}{\underset{j=1}{\sum }}|\beta_{j}|, \end{array}$$

The regularization strength is controlled by the parameter λ, which determines the extent to which penalty terms influence the model. L1-regularization, in particular, promotes sparsity by driving certain coefficients to exactly zero, effectively performing feature selection. In context of our problem, it identifies the most relevant wavelengths (features in the vector ***x***) that contribute to predicting the presence of HCV, ensuring that only the most informative wavelengths are retained in the model.

#### 3.2.3 Random forest

RF is an ensemble learning algorithm that build a “forest” (an ensemble) of uncorrelated decision trees and aggregates their predictions to improve accuracy and robustness. For classification tasks, it determines the final output using majority voting, while for regression, it averages the predictions of individual trees (24, 25).

To build the RF model, the algorithm generates multiple decision trees using bootstrapped samples from the training set. At each node within a tree, a randomly selected subset of features is considered when determining the optimal split. This approach helps to reduce correlation among trees, enhancing the model's generalization ability.

The construction of each decision tree is guided by a criterion that measures the quality of a split. In the case of classification, the algorithm commonly uses the Gini Impurity Index (although alternatives such as Information Entropy exist) to evaluate candidate splits. The Gini Index quantifies the probability of misclassifying a randomly chosen sample at a given node and is defined for binary classification as:


$$\begin{array}{c} G=2p\left(1-p\right), \end{array}$$

where *p* represents the proportion of samples belonging to one of the classes. A pure node, where all samples belong to the same class, has *G* = 0. To determine the optimal split at a given node, the algorithm evaluates all possible feature splits and selects the one that minimizes the weighted Gini Index of the child nodes, given by:


$$\begin{array}{c} G_{\text{split}}=p_{\text{left}}\cdot G_{\text{left}}+p_{\text{right}}\cdot G_{\text{right}}, \end{array}$$

where $p_{left}=\frac{N_{left}}{N}$ and $p_{right}=\frac{N_{right}}{N}$ represent the proportion of samples assigned to the left and right child nodes, respectively (*N* is the total number of samples in the parent node), and *G*_*left*_ and *G*_*right*_ denote the Gini Indices of the child nodes.

RF is widely used for its robustness, ability to handle high-dimensional data, and capability to provide reliable feature importance estimates, making it a powerful tool for various classification and regression tasks.

## 4 Feature importance methods

To enhance the predictive performance of our model combining NIRS and clinical data, we employ two statistical methods to perform feature selection based on feature importance derived from RF. Feature selection is crucial in high-dimensional datasets to reduce redundancy, improve model interpretability, and mitigate overfitting. By leveraging feature importance scores, we systematically identify the most relevant predictors that contribute to the model's performance predicting HCV presence in the serum samples. In this section, first, we describe an agnostic method to estimate the importance: **permutation feature importance** (PFI), and then, two statistical approaches to select features that are relevant based on PFI to refine the feature set, ensuring a robust and efficient predictive framework.

### 4.1 Permutation feature importance

PFI is a model-agnostic technique for quantifying the contribution of individual features to a predictive model's performance. In the context of our RF-based feature selection framework, this method provides an intuitive measure of feature relevance by assessing the impact of randomly shuffling each feature on model accuracy. PFI shuffles the values of a certain feature and see how it affects the performance of the model, then a feature is considered “important” if the performance decreases after shuffling its values (26, 27).

Consider a trained model $\hat{f}$, a feature matrix *X*,a response variable *y* and a performance measure $M\left(y,\hat{f}\right)$, the PFI algorithm can be described as follows:

1. Compute the performance of the orginal model $M_{\text{orig}}=M\left(y,\hat{f}\left(X\right)\right)$ (e.g., mean squared error).
2. For each feature *j* ∈ {1, …, *p*}:
  Repeat *n* times:
  (a) Generate feature matrix $X_{\text{perm}}^{n}$ by permuting feature *j* in the data *X*. This breaks the association between feature *j* and the true outcome *y*.
  (b) Estimate performance $M_{\text{perm}}^{n}=M\left(y,\hat{f}\left(X_{\text{perm}}^{n}\right)\right)$ based on the predictions of the permuted data.
  (c) Calculate permutation feature importance as:
$$FI_{jn}=M_{\text{orig}}^{n}-M_{\text{perm}}^{n}$$

The result is a matrix of permutation importance score *FI* ∈ ℝ^*p*×*n*^.

### 4.2 Feature selection using confidence interval based on PFI

To assess the statistical significance of feature importance scores obtained usign PFI, we estimate **empirical confidence intervals (CIs)** from the resulting distribution. The PFI method generates a matrix *FI* ∈ ℝ^*p*×*n*^, where *p* is the number of features, *n* is the number of permutations (shufflings). Each entry of this matrix, *FI*_*ij*_, represents the importance score of feature *i* in the *j*-th permutation.

#### 4.2.1 Computing empirical confidence intervals

For each feature *i*, we obtain an empirical distribution of importance scores:


$$FI_{i}=\left\{FI_{i1},FI_{i2},\ldots ,FI_{in}\right\}.$$

Using this distribution, we compute the empirical (1 − α) confidence interval based on the percentiles of the observed values:


$$CI_{i}=\left[Q_{\frac{\alpha }{2}}\left(FI_{i}\right),Q_{1-\frac{\alpha }{2}}\left(FI_{i}\right)\right],$$

where *Q*_*p*_(*FI*_*i*_) denotes the *p*-th percentile of the empirical distribution of *FI*_*i*_.

#### 4.2.2 Feature selection criterion

A feature *i* is considered **non-informative** (i.e., unimportant) if its confidence interval contains zero:


$$0\in CI_{i}.$$

This indicates that the feature's importance is not statistically different from 0, and thus, it is discarded from the predictive model.

### 4.3 Permutation importance feature selection

We also consider a modified version the Permutation Importance (PIMP) method proposed by Altmann et al. (28). In this method, to preserve the relation between features, the response variable *y* is permuted. This approach determines the importance of a feature by testing if its importance score is significantly greater than what would be expected by chance. We compare the observed importance of a feature (the Gini importance) with a distribution of importances obtained by randomly permuting *y*. This is a way of creating a null distribution of feature importances, since under the null hypothesis we assume no relation between the features and the response variable.

RF provides a built-in feature importance method based on the contribution of each feature in the trees that form the forest. At each split of a tree, the improvement in the criterion (in our case the Gini Index) is recorded as the split importance for the feature used in that split. For every feature, the split importance from all the nodes are summed up. This gives a measure of how much a feature contributes to the model. These importances are normalized by dividing by the total importance (sum of all the split importances) to ensure they are scaled between 0 and 1. This is the *Gini importance* (GI) for each feature.

The PIMP algorithm can be described as follows. Consider a trained model $\hat{f}$, a feature matrix *X* and a response *y*.

1. Store the GI for all *p* features using $\hat{f}$. These are the baseline importances. *GI*_*b,i*_ is the GI for the feature *i* estimated by the baseline model, i.e. the model trained with the original *X* feature matrix, and the original *y* response variable.
2. Repeat *n* times:
  (a) Permute the response variable *y* to obtain $y_{j}^{*}$, which the permuted version of *y* for the *j*-th permutation.
  (b) Retrain the model using the original feature matrix *X* and the permuted response $y_{j}^{*}$.
  (c) Store the GI of the features for the retrained model. These GIs are estimated under the null hypothesis. *GI*_*H*_0_, *ij*_ is the GI importance for feature *i*, in the *j*-th permutation, under the null hypothesis, i.e., using the permuted response variable $y_{j}^{*}$.
3. For each feature:
  (a) Estimate the empirical cumulative distribution function (ECDF), $\hat{F}\left(x\right)$, of the feature's GI, using *GI*_*H*_0_, *ij*_ which consists of *n* GI values (number of permutations *j* = 1, …, *n*) for the feature *i*, obtained under the null hypothesis *H*_0_.
  (b) Compute the *p*-value as the proportion of permutation-based GI greater or equal to the baseline importance:
$$p-{\text{value}}_{i}=1-\hat{F}\left(x=GI_{b,i}\right)$$

The *p*-value is used to assess whether a feature is independent of the response variable. By comparing it to a significance level (α), we determine whether to reject the null hypothesis. If the *p*-value < α, there is evidence that the feature is associated with the response variable, suggesting its importance in the analysis.

## 5 Experimental setup

Our experimental setup follows a multi-stage machine learning approach to preprocess and analyze the data effectively. The experiment consists of four key steps. The four stages are:

1. **Preprocessing**. We identify the optimal preprocessing technique for NIRS data by evaluating different combinations of methods. To determine the best approach for HCV serum detection, we use a simple LR model. Specifically, we consider two scattering correction methods, MSC and SNV, along with eight different downsampling rates (2,4,8,16, and 25), we also include the option of no preprocessing at all, resulting in a total of 18 NIRS preprocessing combinations.
2. **NIRS wavelength feature selection**. After selecting the optimal preprocessing technique, we apply a feature selection method to identify the most relevant wavelengths for HCV detection. Specifically, we use a L1-penalized LR model, which encourages sparsity by assigning zero coefficients to less important wavelengths, effectively selecting only the most informative features.
3. **RF model and integration with clinical data**. In the third stage, we integrate the selected wavelengths and preprocessing technique with clinical patient data (Table 1) to build a RF model, aiming to enhance the predictive power for HCV detection. Clinical features that are corroboration of the presence of the HCV were discarded (results from biopsy, presence of cyrrhosis, etc). Features with more than 20% of missing values were also discarded. Table 1 shows the clinical features finally used in the model. Missing values for the remaining features were impute using the median value.
4. **Permutation importance statistical feature selection**. In the fourth stage, we use PFI methods to refine the RF model by identifying the most critical features for classification, ensuring that only the most relevant variables contribute to the final prediction.

**Figure 1:**
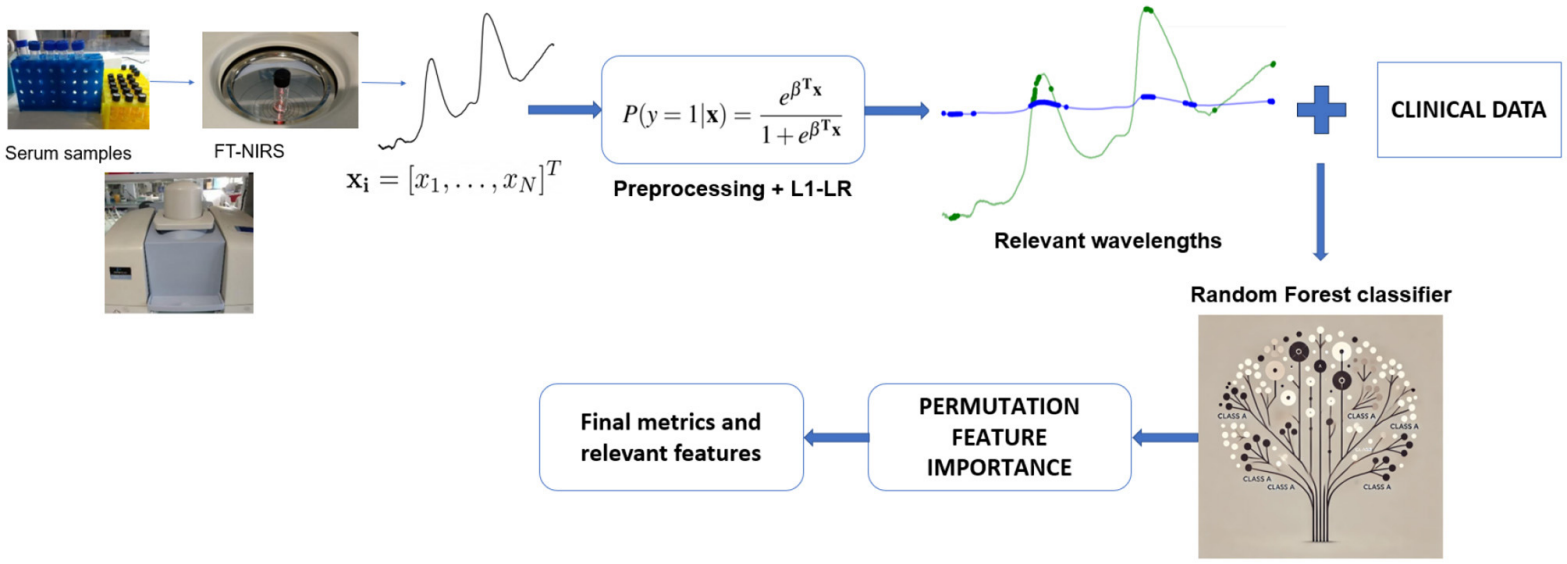
Overview of the multi-stage machine learning pipeline for HCV detection using FT-NIRS data. Serum samples are analyzed generating spectral data. Various preprocessing techniques are evaluated, and the best approach is selected. An L1-penalized LR model is then applied to identify the most relevant wavelengths for HCV detection. These selected wavelengths are combined with clinical data to construct RF classifier. PFI is employed to refine the model by selecting the most critical features. Finally, the optimized RF model is used for classification, and final performance metrics and key features are reported.

Figure 1 illustrates the complete experimental setup.

We split data set keeping 75% of the samples for training set and 25% for test set. In every stage of the experimental setup, we used four-fold cross-validation to tune hyperparameters, preprocessing schemes or model selection. Since multiple serum samples were collected from the same patient at different treatment time points, we adopted a patient-wise approach for both the training-test split and cross-validation. This ensured that all samples from a given patient were assigned to the same subset or fold. Notably, because these samples were taken at different stages of treatment, they could have different target values (i.e., HCV detectable or undetectable).

We used several performance metrics to evaluate the machine learning models. The chosen metrics are accuracy, F1-score, sensitivity, specificity, and area under the curve ROC (AUC-ROC) (29). **Accuracy** is the proportion of correct predictions. **F1-score** is the harmonic mean of precision and recall and is particularly useful in addressing class imbalance. **Sensitivity** (also known as recall or the true positive rate) measures the proportion of actual HCV-positive cases that are correctly identified by the model. **Specificity** (also known as the true negative rate) assesses the proportion of non-HCV cases that are correctly identified by the model. **AUC-ROC** evaluates the model's ability to distinguish between HCV-positive and HCV-negative cases across various decision thresholds. By plotting the true positive rate (sensitivity) against the false positive rate (1-specificity), the AUC-ROC provides a summary measure of the model's discriminatory power, where a higher value indicates better performance. To assess the variability in performance metrics, we employed bootstrap resampling on the test set. Specifically, we generated multiple resampled datasets by sampling with replacement from the original test set, and evaluated the performance of the model on each of these bootstrapped resamples. This approach allowed us to estimate the standard deviation of the performance metrics, providing a measure of their reliability.

## 6 Results

In this section, we present the results of our approach, following the same structure and order as outlined in the Section 5. Each subsection corresponds directly to a specific stage in the experimental process, allowing for a clear and logical progression of the results. This alignment ensures that the reader can easily trace the methodology and understand how the results relate to the steps we followed during the experiment.

### 6.1 Preprocessing

The results demonstrated comparable performance across various preprocessing schemes. To ensure a fair comparison, we selected the two most effective approaches for further stages. Table 2 presents the performance metrics, including AUC, F1-score, accuracy, sensitivity and specificity for these two optimal preprocessing methods.

**Table 2:** Preprocessing NIRS results for the two best schemes.

**Dataset**	**Accuracy**	**f1 score**	**ROC AUC**	**Sensitivity**	**Specificity**
LR sr = 2.0 + SNV	0.5556	0.3333	0.575	0.250	0.800
LR SNV	0.5556	0.3333	0.587	0.250	0.800

LR, logistic regression; sr, downsampling rate; SNV, standard normal variate.

We identified two optimal preprocessing strategies: (1) applying scattering correction using SNV with a downsampling rate of 2.0 and (2) performing SNV correction without any downsampling.

For subsequent analysis, we used two distinct NIRS data representations, each corresponding to one of these preprocessing methods. The first data representation, consisted of input vectors ***x***_2.0+*SNV*_ of 1,500 absorbance values corresponding to 1,500 different wavelengths, obtained using SNV correction with a downsampling rate of 2.0. The second data representation consisted of vectors ***x***_*SNV*_ with 3,001 absorbance values corresponding to 3,001 wavelengths, generated using SNV correction without downsampling.

### 6.2 NIRS wavelength feature selection

**Figure 2:**
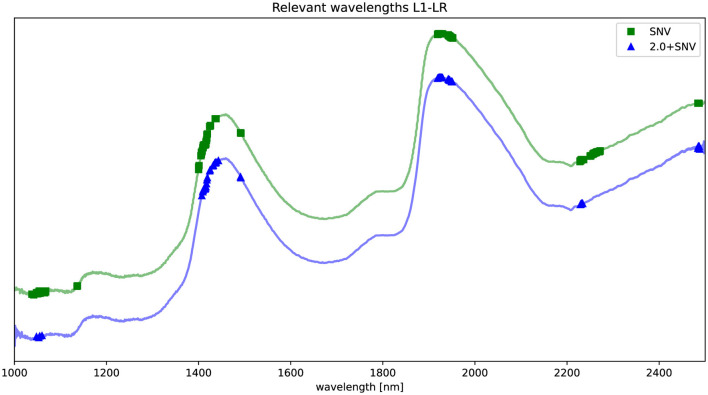
Spectrum preprocessed by SNV (green) and 2.0+SNV (blue). Relevant wavelengths according to L1-regularized LR are highlighted in the figure using a 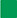 for SNV and 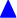 for 2.0+SNV. The NIRS curves are shifted for visualization purposes.

In this section, we present the results of the feature selection process applied to the NIRS data. To identify the most informative wavelengths for predicting HCV presence, we employed an L1-LR model. Figure 2 illustrates the selected wavelengths, marked by dots along the NIRS curve, for both preprocessing techniques: standard normal variate (SNV) and its extended version (2.0+SNV).

The identified key wavelengths are consistent across both preprocessing methods, with clusters centered around 1,100, 1,420, 1,935, 2,230, and 2,485 nm. Notably, the clusters at ~1,100, 1,420, and 1,935 nm align closely with the main absorption peaks of water (30), suggesting a strong relationship between these spectral features and water molecular structure variation in the samples.

After this feature selection stage, the final input vectors representing NIRS data will have the following dimensionality:${\text{x}}_{2.0+SVN}\in {\mathbb{R}}^{55}$,${\text{x}}_{SVN}\in {\mathbb{R}}^{168}$.

In both cases, the feature selection procedure created a drastic dimensionality reduction from 1,500 to 55 and from 3,000 to 168 features.

### 6.3 RF model and integration with clinical data

In this subsection, we present the results for building a RF model to predict the presence of HCV in serum samples combining NIRS data and clinicla data. The data from NIRS was the result of applying feature selection using L1-regularized LR, and keeping the two preprocessing schemes that provided better results, i.e., we are going to use ${\text{x}}_{2.0+SVN}\in {\mathbb{R}}^{55}$, ${\text{x}}_{SVN}\in {\mathbb{R}}^{168}$ and concatenate these vectors with clinical data vector, ${\text{x}}_{CD}\in {\mathbb{R}}^{11}$. So the final input vector that combines both sources of information (NIRS and clinical data) would be:


$${\text{x}}_{2.0+SNV}^{*}=\left[\begin{array}{c} {\text{x}}_{2.0+SNV}\\ {\text{x}}_{CD} \end{array}\right]\in {\mathbb{R}}^{66},\;{\text{x}}_{SNV}^{*}=\left[\begin{array}{c} {\text{x}}_{SNV}\\ {\text{x}}_{CD} \end{array}\right]\in {\mathbb{R}}^{179}$$

Table 3 presents the performance of the RF models . In order to compare if combining NIRS and clinical data improved the prediction capabilities of the model, we also compared with a RF model trained only using clinical data (***x***_*CD*_), denoted as RF^*CD*^, and also two RF model trained only using NIRS data (after feature selection and for both preprocessing schemes), denoted as ${\text{RF}}_{2.0+SNV}^{NIRS}$ and ${\text{RF}}_{SNV}^{NIRS}$. When using only clinical data in the model (RF^*CD*^), we observed solid classification performance (Accuracy = 0.667, F1 score = 0.625, ROC AUC = 0.738). NIRS data alone, preprocessed either with SNV or 2.0+SNV (${\text{RF}}_{2.0+SNV}^{NIRS}$, ${\text{RF}}_{SNV}^{NIRS}$), yields lower performance across all metrics. However, combining clinical data with NIRS features improved model performance.

**Table 3:** Performance results, mean ± standard deviation (accuracy, F1-score, ROC-AUC, Sensitivity and Specificity), for RF models combining NIRS and clinical data (RF*NIRS*+*CD*) for the two preprocessing schemes (SNV and 2.0+SNV).

**Dataset**	**Accuracy**	**f1 score**	**ROC AUC**	**Sensitivity**	**Specificity**
RF^*CD*^	0.67 ± 0.12	0.62 ± 0.12	0.74 ± 0.12	0.62 ± 0.18	0.70 ± 0.15
${\text{RF}}_{2.0+SNV}^{NIRS}$	0.67 ± 0.11	0.57 ± 0.12	0.65 ± 0.13	0.50 ± 0.18	0.80 ± 0.14
${\text{RF}}_{SNV}^{NIRS}$	0.61 ± 0.11	0.53 ± 0.12	0.64 ± 0.15	0.50 ± 0.18	0.70 ± 0.15
${\text{RF}}_{2.0+SNV}^{NIRS+CD}$	**0.72 ± 0.11**	**0.67 ± 0.12**	**0.85 ± 0.11**	**0.63 ± 0.18**	**0.80 ± 0.12**
${\text{RF}}_{SNV}^{NIRS+CD}$	0.67 ± 0.11	0.57 ± 0.11	0.74 ± 0.10	0.50 ± 0.16	0.80 ± 0.15

For comparison we include the results of RF model using only clinical data (RF*CD*, first row) and RF model using only NIRS data (RF*NIRS*). Best results are highlighted in bold font.

The highest accuracy (0.722) and ROC AUC (0.850) were achieved when integrating clinical data with NIRS preprocessed using SNV and a downsampling rate of 2.0 (${\text{RF}}_{2.0+SNV}^{NIRS+CD}$). This combination also improved sensitivity while maintaining high specificity. This combination seemed to maintain the good specificity performance obtained using NIRS data and good sensitivity performance obtained using the clinical data.

These results highlighted the advantage of integrating clinical and NIRS data in RF models, demonstrating that multimodal approaches outperformed models relying on a single data source.

### 6.4 Permutation importance statistical feature selection

In the final stage of the proposed approach, we evaluated the relevance of performing feature selection on the RF models that combined NIRS and clinical data (${\text{RF}}_{2.0+SNV}^{NIRS+CD}$, ${\text{RF}}_{SNV}^{NIRS+CD}$). We compared two permutation importance methods, the first one based on PFI and using confidence intervals (PFI-CI) described in Section 4.2, and the second one based on PIMP and using hypothesis test described in Section 4.3.

Table 4 shows the performance of models after applying feature selection to the best-performing model from the previous stage (${\text{RF}}_{2.0+SNV}^{NIRS+CD}$). The first row shows the results from the model with all the features for sake of comparison.

**Table 4:** Performance results, mean ± standard deviation (accuracy, F1-score, ROC-AUC, Sensitivity and Specificity), for feature selection using the RF models combining NIRS and clinical data (RF*NIRS*+*CD*) for the two permutation methods, PCI-CI and PIMP.

**Dataset**	**Accuracy**	**f1 score**	**ROC AUC**	**Sensitivity**	**Specificity**
* ${\text{RF}}_{2.0+SNV}^{NIRS+CD}$ *	**0.72 ± 0.11**	**0.67 ± 0.12**	**0.85 ± 0.11**	**0.63 ± 0.18**	**0.80 ± 0.12**
${\text{RF}}_{2.0+SNV}^{NIRS+CD}$ PFI-CI	0.56 ± 0.11	0.56 ± 0.11	0.65 ± 0.14	0.63 ± 0.18	0.50 ± 0.16
${\text{RF}}_{2.0+SNV}^{NIRS+CD}$ PIMP	0.67 ± 0.11	**0.67 ± 0.11**	0.76 ± 0.11	**0.75 ± 0.17**	0.60 ± 0.16
${\text{RF}}_{SNV}^{NIRS+CD}$ PFI-CI	0.56 ± 0.11	0.56 ± 0.12	0.71 ± 0.14	0.63 ± 0.16	0.50 ± 0.16
${\text{RF}}_{SNV}^{NIRS+CD}$ PIMP	0.67 ± 0.11	**0.67 ± 0.11**	0.73 ± 0.13	**0.75 ± 0.15**	0.60 ± 0.16

For comparison we include the results of the best RF model using all the features (RF*NIRS*+*CD*_2.0+*SNV*_, first row). Best results are highlighted in bold font.

After feature selection, models using PFI-CI exhibited a drop in accuracy (0.556) and ROC AUC (0.65), suggesting that some important predictive features were removed. However, models using PIMP retained better performance, with accuracy remaining at 0.667 and sensitivity improving to 0.75. They obtained same F1-score as the best model and moderate good results in ROC-AUC. This trend is observed both with and without downsampling, where PIMP consistently outperformed PFI-CI.

Feature selection resulted in models with fewer variables and higher sensitivity, which can be crucial in certain clinical applications. Additionally, this reduction in feature complexity facilitated interpretability, and potentially created a more parsimonious model.

Both ${\text{RF}}_{SNV}^{NIRS+CD}$, ${\text{RF}}_{2.0+SNV}^{NIRS+CD}$ after performing feature selection using PIMP selected the following clinical data GPT, GOT and GGT biomarkers associated with liver function and metabolic processes, suggesting their potential relevance in the prediction of the presence of HCV in the serum sample above the other clinical variables considered.

Table 5 shows the wavelengths selected after performing PIMP for both models. These selected wavelengths correspond to molecular vibrational modes primarily associated with different states of water, including free water, protein-bound water, and water confined in biological matrices. The presence of these water-related bands aligns with findings in aquaphotomics, a field that explores the role of water as a molecular mirror reflecting physiological and biochemical changes.

**Table 5:** Metabolomic assignment of the most significant wavelengths (λ) identified using permutation feature selection approach PIMP from the RF models (${\text{RF}}_{SNV}^{NIRS+CD}$ and ${\text{RF}}_{2.0+SNV}^{NIRS+CD}$).

**RF model and λ**	**Related molecular group**
${\text{RF}}_{SNV}^{NIRS+CD}$ **PIMP**	
1,136.5, 1,413.5, 1,927.0, 2,235.0	1,150 water without active H-bonds (34)
	1,150 free water. Plant, canopy (35)
	1,153 water. Microalga A. erici (39)
	1,160 liquid water near boiling point (36)
	1,160 bulk water (36)
	1,155 free water related with HCV presence (15)
	1,416 free water in human blood (14)
	1,417 C6 (1,413–1,418) free water (S0), quazi-free water molecules. Water molecules confined in the local field of ions. Water with free OH^−^. Positively related with HCV presence (15)
	1,927 protein-bound water in porcine muscle (37)
	1,928 water molecules with one H-bond, S1 (36)
	1,928 primary keratin hydration water in animal stratum corneum of skin (38)
${\text{RF}}_{2.0+SNV}^{NIRS+CD}$ **PIMP**	
1,407.0, 1,410.0, 1,417.0	1,408, 1,416 free water human blood (14)
	1406, 1,417 free water (S0), quazi-free water molecules. Water molecules Confined in the local field of ions. Water with free OH^−^. Positively related with HCV presence (15)

In particular, the wavelengths around 1,150–1,160 nm are linked to free and bulk water, as well as water states associated with biological processes, such as microalgal hydration and liver function. Similarly, the bands near 1,410–1,417 nm correspond to free water in human blood and quasi-free water molecules interacting with ions, which have been positively associated with HCV presence in prior studies. The selection of these wavelengths suggests that water structure alterations in blood could serve as key indicators in the classification task.

Additionally, the wavelengths at 1,927–1,928 nm point to protein-bound water, which has been observed in muscle tissues and keratin hydration processes, further emphasizing the biological relevance of water interactions. Lastly, the bands near 2,252–2,270 nm correspond to lactate and other metabolites, which are known to be involved in metabolic responses, particularly in liver-related conditions.

These findings reinforce the potential of NIRS-based aquaphotomics for capturing subtle biochemical signatures in clinical data. The fact that PIMP consistently selected wavelengths linked to water and metabolite interactions suggests that these spectral features play a crucial role in distinguishing between clinical conditions, possibly reflecting underlying pathophysiological mechanisms.

## 7 Discussion and conclusions

In this study, we explored the integration of NIRS with clinical data and machine learning techniques to improve the detection of HCV in serum samples. Our approach leveraged the non-destructive and rapid diagnostic capabilities of NIRS, combined with the predictive power of machine learning, to create a robust framework for HCV classification. We identified that preprocessing NIRS data using SNV with a downsampling rate of 2.0 provided the best balance between dimensionality reduction and predictive accuracy. This preprocessing step significantly reduced the number of wavelengths from 3,001 to 1,500, while maintaining the integrity of the spectral information. Using L1-regularized LR, we were able to further reduce the dimensionality of the NIRS data, selecting only the most informative wavelengths for HCV detection pointing to potential biomarkers related to disease biochemical mechanisms. This step was crucial in improving model interpretability and reducing overfitting. By combining the selected NIRS wavelengths with clinical data, we observed a significant improvement in model performance. The RF model that integrated both NIRS and clinical data achieved the highest accuracy (0.722) and ROC AUC (0.850), outperforming models that used either NIRS or clinical data alone. We employed PFI methods to refine the feature set, ensuring that only the most relevant features contributed to the final model. The PIMP method, in particular, demonstrated superior performance in maintaining predictive accuracy while reducing the number of features. The selected wavelengths were found to be associated with water molecular structures, such as free water, protein-bound water, and water confined in biological matrices. These findings align with previous studies in aquaphotomics, suggesting that water structure alterations in blood and tissues could serve as key indicators in the classification task.

Beyond classification performance, an important aspect of our approach is the potential for biological insight gained through the relationship between selected wavelengths and specific molecular groups present in serum samples. By linking spectral features to different biochemical components, we can deepen our understanding of the molecular alterations associated with HCV infection, potentially uncovering patterns relevant to disease progression and host response. By emphasizing feature selection and model simplicity, we aimed to enhance generalization capabilities, reducing the risk of overfitting and increasing the potential for real-world applicability.

These findings align with previous studies in NIRS, suggesting that water structure alterations in blood and tissues could serve as key indicators in the classification task (15, 16). We can extract information regarding the overtones of the spectral pattern of water, which mirrors the rest of the components of the solution. This process can be achieved by means of aquaphotomics. Aquaphotomics is the study of this spectral pattern and it provides information regarding the composition of the sample when its solvent is water, increasing the accuracy of the results (6). However, our study extends these findings by integrating clinical data, which significantly enhances the predictive power of the model. This multimodal approach aligns with the growing trend in biomedical research, where combining multiple data sources (e.g., imaging, genomics, and clinical data) has been shown to improve diagnostic accuracy and provide deeper insights into disease mechanisms.

The biological rationale behind NIRS is that it detects molecular overtones and combination bands related to O–H, N–H, and C–H bonds, which are abundant in biological samples due to the presence of water, proteins, lipids, and small molecules. Water constitutes 92%–94% of serum, and the O–H bonds established dominate NIR spectrum (31). From a clinical standpoint, HCV infection leads to well-characterized biochemical alterations in the serum, including changes in hepatic protein synthesis, lipid metabolism, and immune activation. These alterations influence serum composition, which in turn can affect how water molecules interact with surrounding solutes. Even subtle changes in the serum elements can affect the water O–H bonding network provoking shifts in NIR absorption. In this context, the hydrogen bonding environment of water, reflected in specific NIRS absorption regions, may be sensitive to the presence of inflammation-related proteins (TNF-alpha, IL 6 and others), viral RNA, or metabolic by-products. While we do not claim direct spectral detection of HCV particles, the infection-associated serum milieu likely generates a distinguishable NIRS fingerprint. Similar applications of NIRS have been reported in diabetes and oncology, where complex systemic changes alter serum spectra measurably (14, 15, 32).

The potential influence of treatment regimen on observed spectral differences was carefully considered. All patients included in the study, both those who cleared HCV and those who did not, received the same treatment regimen consisting of peginterferon plus ribavirin. Therefore, the treatment exposure was uniform across both groups, eliminating ribavirin as a differential factor in the spectral analysis. Given this uniform exposure, any discrimination achieved by our NIRS-based model reflects biological differences associated with HCV positivity rather than treatment-induced spectral changes. While ribavirin cannot be ruled out as a background signal it is not likely to be a confounding factor in the classification task.

NIRS offers a non-invasive, safe, and rapid diagnostic method, which is particularly advantageous in clinical settings where time and sample preservation are critical, and cost-efficient. FT-NIRS equipment typically involves a one-time investment ranging from €20,000–€40,000, which is comparable to PCR systems. However, NIRS testing requires no reagents or consumables, leading to a much lower per-sample cost, estimated at <€1, compared to €15–€40 for PCR or ELISA (19, 33). Additionally, NIRS enables rapid analysis (under 1 min per sample), reducing labor and operational costs. These characteristics suggest that NIRS could serve as a cost-effective, high-throughput screening tool, especially in settings with limited laboratory infrastructure.

Our approach effectively reduces the high dimensionality of NIRS data, making it more manageable for machine learning algorithms while retaining the most informative features. By combining NIRS with clinical data, we leverage complementary information, leading to improved diagnostic accuracy. This approach is particularly valuable in complex diseases like HCV, where multiple biomarkers and clinical factors contribute to disease progression. The use of feature selection techniques, such as L1-regularized LR and permutation importance, enhances the interpretability of the model. This is crucial for clinical applications, where understanding the underlying biological mechanisms is as important as achieving high predictive accuracy.

As clinicians entering a technology-driven diagnostic space, we recognize the importance of reproducibility and bias mitigation. Accordingly, all serum samples were processed under standardized conditions: uniform sample volume, temperature, and cuvette type were used for all acquisitions. The NIRS device was calibrated routinely using internal standards, and the acquisition software was automated, minimizing inter-operator variability. Importantly, our machine learning pipeline included data normalization, cross-validation, and outlier detection steps to enhance robustness and reduce susceptibility to noise or batch effects. Nonetheless, we acknowledge that device-specific calibration protocols and sample handling consistency are essential for future clinical translation, and we outline these as limitations and areas for standardization in future studies.

One of the major limitations of our study is the relatively small sample size, consisting of 137 serum samples from 38 patients. This constrained cohort reduces the statistical power of our findings and limits generalizability. The limited sample size reflects the complex logistics and strict biosafety protocols involved in acquiring and analyzing NIRS data from HCV-infected serum samples, which adds significant challenges to expanding the dataset. However, we carefully designed the validation process to construct appropriate validation datasets and experimental setup to provide unbiased performance estimates and mitigate the risk of overfitting. This work should be viewed as a proof of concept, demonstrating demonstrate the feasibility of leveraging NIRS data in combination with clinical or biochemical information for diagnostic modeling. While our preliminary results are encouraging, especially in terms of specificity, they require validation in larger and more diverse populations. Future studies should be prospectively designed with adequate power and expanded data inputs (including imaging, genetic, or longitudinal clinical data) to fully realize the potential of this integrative diagnostic framework. In the current state, the sensitivity of the presented approach (75%) remains below the threshold typically required for a clinical diagnostic tool (e.g. compared to PCR), where higher sensitivity is essential to minimize the risk of missed diagnoses. As a next step, we aim to investigate cost-sensitive learning approaches, which assign higher penalties to misclassifying minority or high-risk classes (e.g., false negatives in disease detection), thereby shifting the model's focus toward improved sensitivity. Combined with advanced ensemble methods, this strategy may help achieve performance levels more suitable for clinical applications.

Future research should focus on validating our approach on larger and more diverse datasets, including samples from different populations and disease stages. This will help ensure the generalizability and robustness of the model. Based on our preliminary power calculations, ~200 samples per group would be required to detect a 10% difference in sensitivity (from 80 to 90%) with 80% power and a significance level of 0.05 Exploring more advanced machine learning techniques, such as deep learning or ensemble methods, could further improve model performance, particularly in terms of sensitivity and specificity. Future studies should aim to translate this approach into real-world clinical settings, where rapid and accurate HCV diagnostics are urgently needed. This will require collaboration with healthcare providers and regulatory bodies to ensure the practical applicability of the model. Further exploration of aquaphotomics and its role in biomarker discovery could uncover new insights into the molecular mechanisms underlying HCV infection. This could lead to the identification of novel therapeutic targets and diagnostic markers.

To move this proof-of-concept toward clinical application, a structured translational pathway is essential. The next phase should involve validation in larger, prospectively collected, and ideally multi-center cohorts to ensure that the diagnostic model is robust. Standardization of key steps–such as serum collection, storage, NIRS acquisition, and spectral preprocessing–will be necessary to ensure reproducibility and alignment with clinical laboratory standards (e.g., CLIA, CAP). Performance benchmarks for clinical readiness should target sensitivity and specificity above 90%, particularly in screening contexts where avoiding false negatives is critical. Beyond technical performance, successful implementation will require seamless integration into clinical workflows. This includes developing decision support tools compatible with electronic health record systems, and ensuring minimal disruption to clinician routines. Health economic evaluations will also be necessary to assess cost-effectiveness compared to conventional diagnostics (e.g., PCR, HPLC), accounting for equipment costs, throughput, and potential downstream savings from earlier intervention, together with the reduction of biological and chemical hazards and operators safety risk. Importantly, this work also highlights the novel integration of aquaphotomics principles–focused on water absorbance features as biomarkers of systemic change–into a machine learning framework for serum-based diagnostics. We see this as a foundational step toward a broader platform that leverages NIRS and aquaphotomics not only for detecting infectious diseases, but also for addressing more complex clinical challenges such as risk stratification in HCC using our data available right now. Future research will focus on expanding the spectral and clinical data dimensions and validating this integrative approach in prospective studies designed to evaluate both diagnostic performance and clinical impact.

In conclusion, our study demonstrates the potential of combining NIRS with clinical data and machine learning for the rapid and non-invasive detection of HCV in serum samples. By leveraging the strengths of NIRS and machine learning, we significantly reduced data dimensionality while maintaining high predictive accuracy. While our method is designed for qualitative classification of HCV status, the NIRS technique is inherently sensitive to the concentration of numerous serum constituents, including those affected by treatment and individual metabolic variability. This underscores the need for further studies to standardize sample collection and consider potential confounders due to differing analyte concentrations. The integration of clinical data further enhanced model performance, highlighting the value of multimodal approaches in medical diagnostics. While limitations such as sample size and suboptimal sensitivity (75%) must be addressed, this work lays a strong foundation for future research. Importantly, it serves as a proof of concept for applying NIRS-derived spectral fingerprints as biochemical phenotypes, which could support more complex tasks–such as predicting progression to HCC in future translational studies. With further validation and refinement, this approach could evolve into a cost-effective and scalable diagnostic tool, particularly valuable in resource-limited settings.

## Data Availability

The original contributions presented in the study are included in the article/supplementary material, further inquiries can be directed to the corresponding author.
